# Occurrence of corneal sub-epithelial microneuromas and axonal swelling in people with diabetes with and without (painful) diabetic neuropathy

**DOI:** 10.1007/s00125-023-05945-0

**Published:** 2023-06-10

**Authors:** Eva Sierra-Silvestre, Ricardo J. Andrade, Luisa H. Colorado, Katie Edwards, Michel W. Coppieters

**Affiliations:** 1 Menzies Health Institute Queensland, Griffith University, Brisbane, QLD Australia; 2 School of Health Sciences and Social Work, Griffith University, Brisbane, QLD Australia; 3 Amsterdam Movement Sciences – Musculoskeletal Health Program, Faculty of Behavioural and Movement Sciences, Vrije Universiteit Amsterdam, Amsterdam, the Netherlands; 4 Movement – Interactions – Performance (MIP), Nantes University, Nantes, France; 5 Centre for Vision and Eye Research, School of Optometry and Vision Science, Queensland University of Technology, Brisbane, Australia

**Keywords:** Confocal microscopy, Diabetes, Neuroma, Neuropathy, Pain

## Abstract

**Aims/hypothesis:**

Non-invasive in vivo corneal confocal microscopy is gaining ground as an alternative to skin punch biopsy to evaluate small-diameter nerve fibre characteristics. This study aimed to further explore corneal nerve fibre pathology in diabetic neuropathy.

**Methods:**

This cross-sectional study quantified and compared corneal nerve morphology and microneuromas in participants without diabetes (*n*=27), participants with diabetes but without distal symmetrical polyneuropathy (DSPN; *n*=33), participants with non-painful DSPN (*n*=25) and participants with painful DSPN (*n*=18). Clinical and electrodiagnostic criteria were used to diagnose DSPN. ANCOVA was used to compare nerve fibre morphology in the central cornea and inferior whorl, and the number of corneal sub-epithelial microneuromas between groups. Fisher’s exact tests were used to compare the type and presence of corneal sub-epithelial microneuromas and axonal swelling between groups.

**Results:**

Various corneal nerve morphology metrics, such as corneal nerve fibre length and density, showed a progressive decline across the groups (*p*<0.001). In addition, axonal swelling was present more frequently (*p*=0.018) and in higher numbers (*p*=0.03) in participants with painful compared with non-painful DSPN. The frequency of axonal distension, a type of microneuroma, was increased in participants with painful and non-painful DSPN compared to participants with diabetes but without DSPN and participants without diabetes (all *p*≤0.042). The combined presence of all microneuromas and axonal swelling was increased in participants with painful DSPN compared with all other groups (*p*≤0.026).

**Conclusions/interpretation:**

Microneuromas and axonal swelling in the cornea increase in prevalence from participants with diabetes to participants with non-painful DSPN and participants with painful DSPN.

**Graphical Abstract:**

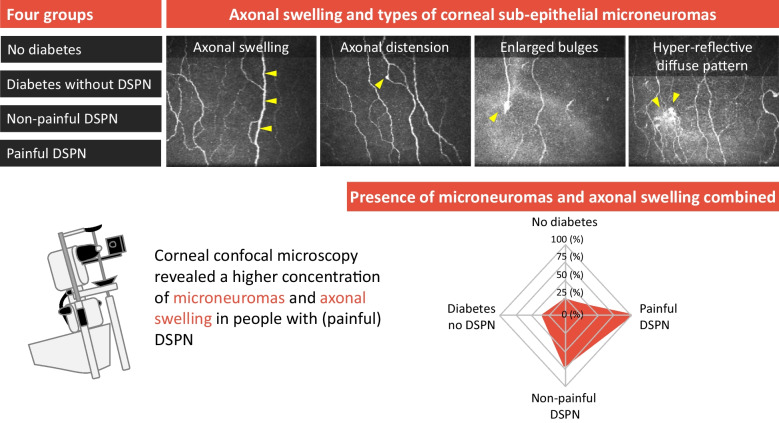

**Supplementary Information:**

The online version of this article (10.1007/s00125-023-05945-0) contains peer-reviewed but unedited supplementary material.


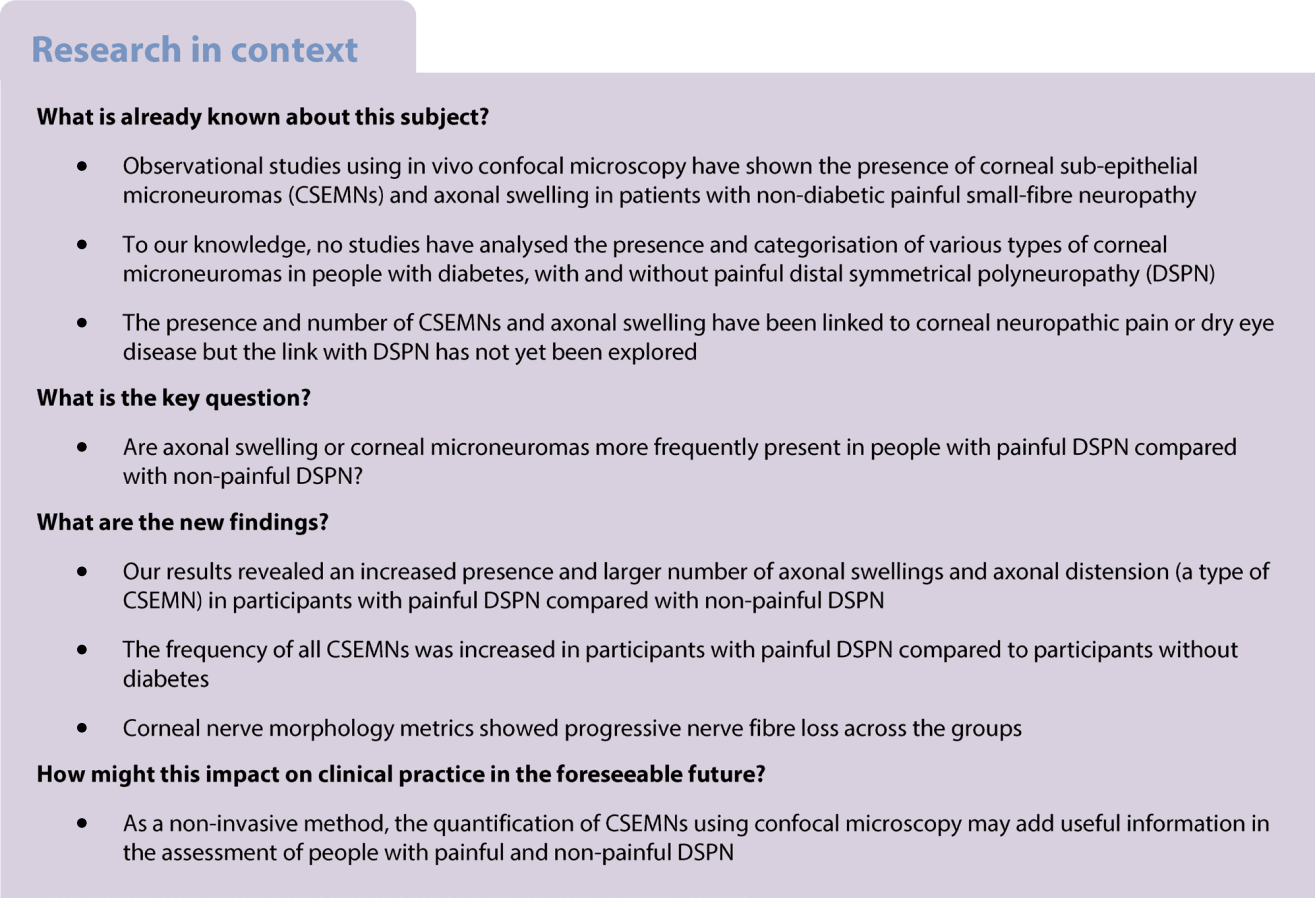


## Introduction

Diabetes and its associated complications are a serious concern worldwide. It is anticipated that one-third of the global population will have diabetes by 2050 [1]. Half of the people who have diabetes develop neuropathy [1], and 15–25% have painful neuropathy [2]. The most prevalent form of diabetic neuropathy is distal symmetrical polyneuropathy (DSPN). Why some patients develop neuropathic pain, while others with a similar degree of neuropathy do not, is still not clearly understood [3].

Although the mechanisms involved in the development of non-painful and painful DSPN remain uncertain [2], several peripheral nerve features have been suggested as biomarkers of painful neuropathy, such as axonal swelling or nerve fibre loss [4]. In vivo corneal confocal microscopy (IVCM) has emerged as a non-invasive alternative method for imaging structural peripheral nerve features rapidly and accurately [5]. Use of IVCM has revealed that corneal nerve fibre length and density at the central corneal sub-basal plexus are reduced in people with diabetes, both with and without DSPN [6]. This deterioration appears to be more pronounced at the distal end of the corneal sub-basal plexus, i.e. at the inferior whorl [7]. Moreover, axonal swelling is more frequently observed in people with diabetes regardless of the presence of neuropathy [8]. Some authors revealed that the extent of corneal nerve fibre deterioration is greater in people with painful compared with non-painful DSPN [6, 7], but these findings are not conclusive [2].

Corneal sub-epithelial microneuromas (CSEMNs) are another example of structural nerve features that arise when mechanical trauma to corneal nerves occur (e.g. refractive surgery) or in systemic diseases (e.g. diabetes) [9]. CSEMNs include axonal distension, enlarged bulges and hyper-reflective diffuse patterns [10]. They are common in processes of abnormal nerve regeneration, which occur in diabetes and DSPN. To our knowledge, only one study [11] has explored CSEMNs in diabetes, showing greater numbers of CSEMNs in participants with diabetes compared to participants without diabetes. This study [11] also found a correlation between CSEMN frequency and poorer measures of glucose control, which have been associated with painful DSPN [12]. However, DSPN was not specifically investigated as participants with painful vs non-painful DSPN were not included.

In addition to diabetes, CSEMNs have been explored in patients with ocular conditions, such as dry eye and corneal neuropathic pain [13]. However, the findings are conflicting. CSEMNs have been observed in patients with dry eyes who had symptoms of neuropathic pain [14], but a later study found no correlation between the frequency of CSEMNs and corneal neuropathic pain [15]. Moreover, some features of CSEMNs may be present in people with healthy corneas (e.g. hyper-reflective diffuse patterns) [16]. Quantifying and categorising CSEMNs may provide further insight into the pathophysiology of painful and non-painful DSPN. The aims of this study were to: (1) compare the corneal nerve morphology in the central cornea and the inferior whorl between participants without diabetes, participants with diabetes but without DSPN, participants with non-painful DSPN, and participants with painful DSPN; and (2) compare the presence and attributes of microneuromas and axonal swelling in these populations.

## Methods

This study was part of a broader research initiative (The DIAbetic NEuropathy (DIANE) Project) in which nerve function and morphology were comprehensively assessed to better understand DSPN. The somatosensory profiles have been published elsewhere [17]. The study was approved by the Ethics Committee of Griffith University (2018/669) and the Queensland University of Technology (1800001224). All participants provided written consent prior to the study, and were recruited between August 2019 and December 2020.

### Participants

Participants were above 18 years of age with or without diabetes (type 1 or type 2). People with diabetes were eligible if they had no DSPN, non-painful DSPN or painful DSPN. DSPN could be present in the lower limbs only, or in lower and upper limbs.

Exclusion criteria were: the presence of conditions that restrict or influence IVCM (e.g. positive corneal staining corresponding to a score of less than 2 on the Efron scale [18]; cataract surgery in the past year; use of rigid contact lenses; medication for glaucoma; or laser eye surgery). Further exclusion criteria were: conditions that may mimic DSPN (e.g. hypothyroidism, vitamin B_12_ deficiency, degenerative disc disease or nerve root compression); unilateral symptoms indicative of neuropathy (e.g. known history of lumbar discogenic disease, nerve root compression or history of compressive mononeuropathies); trauma-related nerve injuries; self-reported psychiatric disorders; fibromyalgia; irritable bowel syndrome; chronic fatigue syndrome; complex regional pain syndrome; and a history of malignancy or chemotherapy.

### Groups and classification criteria

Participants were allocated to one of four groups: (1) participants without diabetes; (2) participants with diabetes but without DSPN; (3) participants with non-painful DSPN; and (4) participants with painful DSPN.

To confirm diabetes, the HbA_1c_ level determined using the Afinion test system (Abbott, USA) had to be ≥42 mmol/mol (≥6%). For participants without diabetes, the HbA_1c_ level had to be <42 mmol/mol (<6%). To confirm the presence of DSPN, the following criteria were used: (1) a bilateral symmetrical presentation of symptoms and signs indicative of DSPN; and (2) abnormal fibular motor nerve conduction. The clinical assessment included testing sensitivity using a 10g monofilament for the presence of signs of neuropathy [19]. To assess the typical distribution of DSPN, participants marked their symptoms, such as numbness, tingling and pain, on a body chart. Electrodiagnostic tests were performed using a neurodiagnostic system (Sierra Summit, Cadwell, USA) according to recommendations by the American Academy of Neurology, the American Association of Electrodiagnostic Medicine and the American Academy of Physical Medicine and Rehabilitation [20]. DSPN was confirmed based on a reduced conduction velocity of the fibular motor nerve (<42 m/s) [21]. People without diabetes were excluded if they had an abnormal electrodiagnosis. Sural sensory, fibular motor, tibial motor, median sensory and motor, and ulnar motor nerves were evaluated for descriptive purposes.

The participants with DSPN were dichotomised into non-painful DSPN and painful DSPN based on the average (mean) pain intensity score over the week prior to the assessment measured using an 11-point numerical rating scale (NRS), where 0 represents no pain and 10 represents the worst possible pain imaginable [22]. If the pain intensity was ≥4, participants were allocated to the painful DSPN group. If the pain intensity was <4, participants were allocated to the non-painful DSPN group [23]. A score of 4 using the NRS is considered the optimal cut-off score to differentiate between participants with DSPN with no or mild pain and participants with moderate or severe pain [22]. Participants with DSPN in the lower and upper limbs were allocated to the painful DSPN group if DSPN was considered painful in either the lower or upper limbs or both. Although use of this cut-off to differentiate painful from non-painful DSPN is common [7, 22–24], we also performed a sensitivity analysis in which only pain-free participants (score on NRS = 0) were included in the non-painful DSPN group.

### Additional participant characteristics

A standardised patient assessment was performed to collect additional data, including sex, age, BMI, ethnicity, type of diabetes, years with diabetes, years with DSPN and medication. Current pain intensity, and least, worst and average pain intensity during the preceding week were measured using the 11-point NRS. The Michigan Neuropathy Screening Instrument [25] was used to further evaluate the presence of DSPN. It is a self-administered questionnaire consisting of 15 questions on foot sensation and pain, numbness and temperature sensitivity. A score of 4 or higher indicates diabetic neuropathy, with higher scores indicating more neuropathic symptoms. Health-related quality of life was evaluated using the EuroQol questionnaire with five dimensions and five severity levels (EQ-5D-5L) [26, 27] to obtain an overall index score based on mobility, self-care, usual activities, pain/discomfort and anxiety/depression. This index ranges from less than 0 to 1 (the value of full health), with higher scores indicating higher health-related quality of life.

### Corneal confocal microscopy parameters

Participants underwent an examination of the sub-basal plexus using a Rostock Cornea Module III tomograph (Heidelberg Engineering, Germany), as described previously [28], in the same week as the diagnostic assessment. As diabetes has a similar impact on corneal nerve fibres in both eyes [29], only the right eye was assessed. An experienced examiner evaluated the central sub-basal nerve plexus and the inferior whorl region of the right eye after instillation of topical anaesthetic (benoxinate hydrochloride 0.4%) and viscous eye gel. An investigator blinded to the group allocation selected five to eight images (400 × 400 µm) with non-overlapping areas for the centre of the cornea, and one image for the inferior whorl region. The image selection criteria were based on image quality and focus.

Corneal nerve fibre density (CNFD), corneal nerve branch density (CNBD), corneal nerve fibre length (CNFL), corneal nerve fibre area (CNFA), corneal nerve total branch density (CTBD), corneal nerve fibre width (CNFW), corneal nerve fractal dimension (CNFractalDimension) and inferior whorl length (IWL) were automatically quantified using ACCMetrics software (University of Manchester, UK) (see electronic supplementary material [ESM] Methods, Corneal confocal microscopy parameters) [30]. In addition, a combination of metrics was used, including the ratio of CNFL/CNFractalDimension to adjust for the degree of nerve loss [31], the average (mean) nerve fibre length (ANFL, [CNFL + IWL]/2) (mm/mm^2^) and total nerve fibre length (TNFL, CNFL + IWL) (mm/mm^2^) [7].

**Fig. 1:**
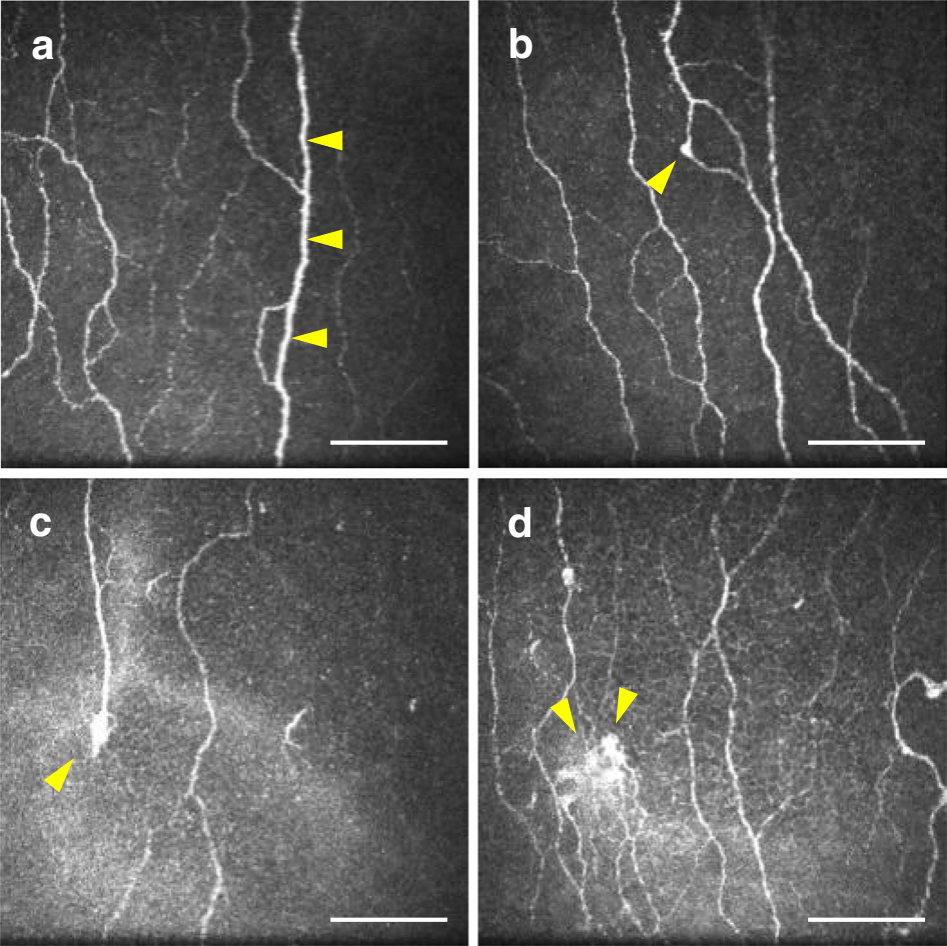
Types of corneal sub-epithelial microneuromas and axonal swelling. Yellow arrowheads show the location of neuromas, except for (**a**), in which they indicate swelling of the nerve fibre, and in (**d**), in which they indicate a large hyper-reflective diffuse pattern. Scale bar, 100 µm

Axonal swelling is defined as thickening of the corneal fibre along its length to more than double the axon diameter [32]. An example of axonal swelling is shown in Fig. 1. The presence (i.e. yes/no) and frequency (i.e. count) of axonal swelling was quantified manually as described in previous publications [11, 14]. CSEMNs were identified in the central cornea from the selected images, and were manually categorised as described previously [16] as axonal distension, enlarged bulges and hyper-reflective diffuse patterns. A representation of these CSEMNs is shown in Fig. 1. Axonal distension is defined as the presence of a round, localised thickening in a nerve fibre. Enlarged bulges are defined as fusiform bulges that are bigger in size than the axonal distension. Hyper-reflective diffuse patterns are defined as bright areas with undefined shapes. If the same microneuroma was present in more than one frame of the selected images, it was considered as a count of one.

### Statistical analysis

The statistical analysis was performed using RStudio version 3.6 [33]. The normality of the distribution of the data was checked using the Shapiro–Wilk test, and the homogeneity of variances across groups was checked using Levene’s test. Data are reported as means ± SD if normally distributed or medians (IQR) if not normally distributed. The comparison between groups was performed using ANCOVA with one between-group factor with four levels (groups), while controlling for age. Post hoc tests using the Bonferroni–Holm correction were applied to adjust *p* values for multiple comparisons. Possible covariates, such as HbA_1c_, duration of diabetes and years with diabetes, were explored but could not be included in the analysis due to violation of an ANCOVA prerequisite, i.e. lack of independence of the covariate with the independent variable (i.e. groups) (see ESM Methods).

Categorical variables were compared using Fisher’s exact test. Pairwise tests of independence for multiple comparisons were performed if the Fisher’s exact test was significant. Fisher’s exact tests were performed using the Bonferroni–Holm correction to adjust *p* values for multiple comparisons.

### Sample size calculations

The study of axonal swelling and CSEMNs is exploratory, hence the sample size was calculated based on previously reported findings regarding CNFL [34]. A difference in scores of 2.7±0.9 mm/mm^2^ has been shown to be clinically significant between participants without diabetes and participants with DSPN [35]. With a power of 0.80 and a two-tailed α of 0.05, a minimum sample size of 16 participants per group was required. As each participant needed to attend assessments at various locations and on different days, a dropout rate of approximately 10% was considered likely, as for previous studies from the DIANE Project [17]. Therefore, the required sample size was at least 18 participants per group.

## Results

### Participants and groups

**Fig. 2:**
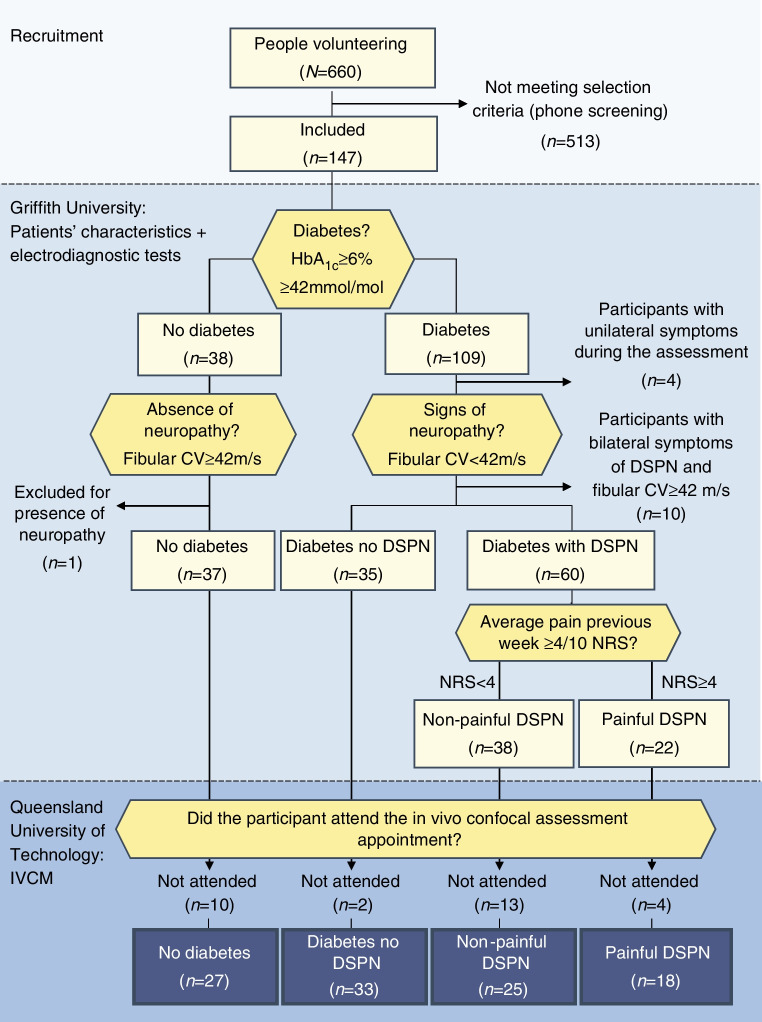
Flow chart of participants. CV, conduction velocity

**Fig. 3:**
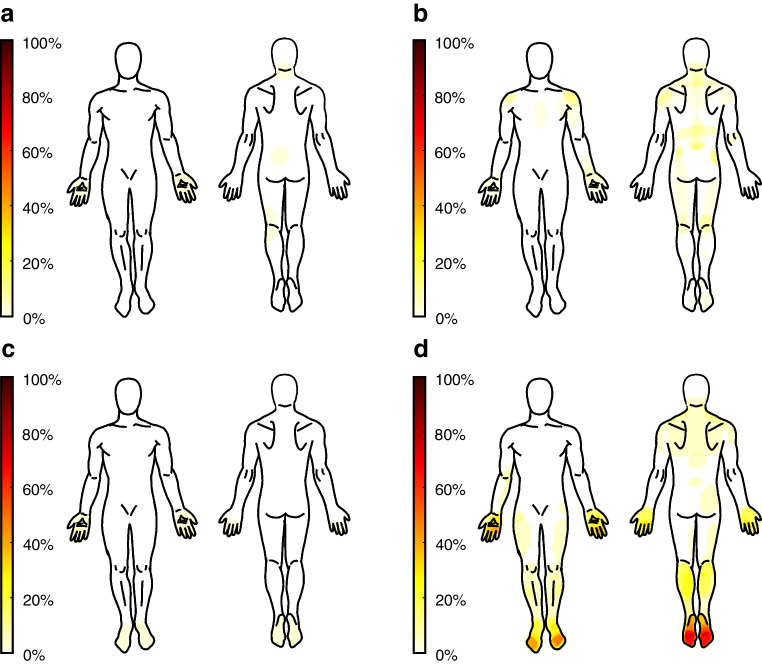
Heat maps represent the location of pain: (**a**) participants without diabetes; (**b**) participants with diabetes but without DSPN; (**c**) participants with non-painful DSPN; (**d**) participants with painful DSPN. The heat bars represent the percentage of participants in each group. Because a pain intensity score on the NRS ≥4 was the criterion for painful neuropathy, some mild to moderate pain may have been experienced in the non-painful DSPN group

Of the 660 people who volunteered for the study, 103 met all selection criteria to participate in the study (participants without diabetes, *n*=27; participants with diabetes but without DSPN, *n*=33; participants with non-painful DSPN, *n*=25; participants with painful DSPN, *n*=18). Figure 2 illustrates the recruitment and enrolment of the participants into the study. The demographic characteristics and the results of the electrodiagnostic tests for group classification are reported in Table 1. The distribution of pain for the various groups is summarised in Fig. 3. The medication used is shown in Table 2. Eleven participants were included in the pain-free group (score on NRS = 0) for the sensitivity analysis.

**Table 1:** Overview of demographic characteristics

	No diabetes (*n*=27)	Diabetes but no DSPN (*n*=33)	Non-painful DSPN (*n*=25)	Painful DSPN (*n*=18)	*p* value	Pairwise comparisons
Age (years)	48.9±16.0	46.6±17.1	63.3±8.4	59.4±8.5	<0.001	1−2, 2−3, 2−4
Female	14 (52)	15 (45)	5 (20)	9 (50)	0.082	
BMI (kg/m^2^)	24.8±4.3	27.5±5.1	30.2±5.0	38.3±8.6	<0.001	1−3, 1−4, 2−4, 3−4
Ethnicity						
Aboriginal	0 (0)	0 (0)	2 (8)	1 (6)		
Asian	4 (15)	7 (21)	0 (0)	1 (6)		
Mixed	1 (4)	2 (6)	1 (4)	0 (0)		
White	22 (81)	22 (67)	22 (88)	14 (78)		
Not reported	–	2 (6)	–	2 (11)		
Type 2 diabetes	–	16 (48)	20 (80)	14 (78)	0.016	
Pain intensity (NRS score)						
Least pain last week	0.3±0.7	0.4±0.9	0.6±1.0	3.3±2.0	<0.001	1−4, 2−4, 3−4
Worst pain last week	1.6±2.1	1.5±1.7	2.2±2.2	6.8±1.3	<0.001	1−4, 2−4, 3−4
Average pain last week	0.8±1.3	1.0±1.3	1.4±1.2	5.5±1.4	<0.001	1−4, 2−4, 3−4
Current pain	0.3±0.8	1.2±1.8	0.9±1.4	4.2±2.1	<0.001	1−4, 2−4, 3−4
Duration of diabetes (years)	–	12.8±10.3	12.9±10.0	14.6±10.4	0.59	
Duration of DSPN (years)	–	–	3.8±4.4	4.8±5.0	0.50	
HbA_1c_ (%)	5.4±0.3	6.6±0.8	8.3±1.6	8.8±1.4	<0.001	1−2, 1−3, 1−4, 2−3, 2−4
HbA_1c_ (mmol/mol)	35.4±3.4	48.7±8.3	64.5±17.9	73.2±15.4		
BP (mmHg)	120±14.6/73.0±8.1	122.5±13.9/75.7±8.3	138.4±24.3/80.8±14.1	141.6±13.8/86.5±11.4	<0.001	1−3, 1−4, 2−3, 2−4
MNSI score	1.2±1.1	1.7±1.5	5.0±2.4	7.7±2.3	<0.001	1−3, 1−4, 2−3, 2−4, 3−4
EQ-5D-5L score	1±0.1	0.9±0.1	0.9±0.1	0.8±0.1	<0.001	1−4, 2−4, 3−4
Electrodiagnostic tests						
Sural SNAP (µV)	9.2±6.2	8.2±3.7	4.2±1.8	4.4±1.8	<0.026	1−3, 1−4, 2−3, 2−4
Sural SCV (m/s)	42.5±8.1	39.5±9.3	34.3±9.9	35.3±11.7	<0.027	1–3
Fibular MCV (m/s)	54.8±6.4	52.1±9.9	39.3±6.0	39.8±7.8	<0.001	1−3, 1−4, 2−3, 2−4
Tibial CMAP (mV)	11.9±3.6	9.2±4.0	4.6±2.8	3.3±2.7	<0.022	1−2, 1−3, 1−4, 2−3, 2−4
Tibial MCV (m/s)	52.1±4.8	49.3±7.8	42.2±8.6	40.9±10.2	<0.0035	1−3, 1−4, 2−3, 2−4
Median CMAP (mV)	7.5±2.3	5.3±1.4	5.0±1.6	3.9±1.6	<0.019	1−2, 1−3, 1−4
Median MCV (m/s)	74.1±7.1	70.4±7.6	64.9±8.0	61.5±7.1	<0.014	1−3, 1−4, 2−4
Median SNAP (µV)	33.8±19.1	19.3±14.5	6.7±4.5	6.0±3.0	<0.018	1−2, 1−3, 1−4, 2−3, 2−4
Median SCV (m/s)	44.5±6.9	47.3±6.7	36.7±7.9	34.5±8.2	<0.046	1−3, 1−4, 2−3, 2−4
Ulnar SNAP (µV)	33.3±17.8	24.2±14.7	11.3±5.0	9.9±5.1	<0.0060	1−3, 1−4, 2−3, 2−4
Ulnar SCV (m/s)	49.5±8.6	50.6±5.8	42.3±10.6	34.6±12.3	<0.0020	1−4, 2−4

Continuous data are means ± SD and were analysed by one-way ANOVA with post hoc tests using the Bonferroni–Holm correction (pairwise differences). Categorical data are *n* (%)

The maximum score on the NRS is 10. BP was measured in the supine position. Due to a technical error, the fibular CMAP could not be determined accurately, and the data have therefore been omitted

Pairwise differences indicate significant *p* values between group pairs as indicated in the final column: (1) participants without diabetes; (2) participants with diabetes but without DSPN; (3) participants with non-painful DSPN; (4) participants with painful DSPN. For each variable, the largest, but still significant, *p* value is provided

CMAP, compound muscle action potential; MCV, motor conduction velocity; MNSI, Michigan Neuropathic Screening Instrument; SCV, sensory conduction velocity; SNAP, sensory nerve action potential

**Table 2:** Overview of medication use in participants without diabetes, participants with diabetes but without DSPN, participants with non-painful DSPN and participants with painful DSPN

	No diabetes (*n*=27)	Diabetes but no DSPN (*n*=33)	Non-painful DSPN (*n*=25)	Painful DSPN (*n*=18)	*p* value	Pairwise comparisons
Hypolipidaemic medication	2 (7)	9 (27)	11 (44)	10 (56)	0.016	1−3, 1−4
Antihypertensive medication	3 (11)	12 (36)	15 (60)	10 (56)	<0.001	1−3, 1−4
Glaucoma medication	0 (0)	0 (0)	1 (4)	0 (0)	0.42	
Antiasthmatic medication	0 (0)	1 (3)	0 (0)	3 (17)	0.023	
Antidepressant medication						
Tricyclic antidepressants	0 (0)	0 (0)	0 (0)	1 (6)	0.17	
SNRI (duloxetine)	0 (0)	0 (0)	0 (0)	2 (11)	0.029	
SSRI (citalopram)	1 (4)	1 (3)	1 (4)	1 (6)	1	
NDRI (methylphenidate)	0 (0)	0 (0)	1 (4)	0 (0)	0.42	
Anxiolytic medication						
Benzodiazepines (diazepam)	0 (0)	0 (0)	0 (0)	0 (0)	–	
Gabapentinoids						
Pregabalin	1 (4)	2 (6)	2 (8)	2 (11)	0.8	
Non-opioid analgesics						
NSAIDs (ibuprofen)	1 (4)	3 (9)	2 (8)	4 (22)	0.27	
Opioid analgesics						
Moderate (codeine)	0 (0)	0 (0)	0 (0)	0 (0)	–	
Antagonists (naloxone)	0 (0)	0 (0)	0 (0)	1 (6)	0.17	

Data are *n* (%)

Pairwise differences indicate significant *p* values between group pairs as indicated in the final column: (1) participants without diabetes; (2) participants with diabetes but without DSPN; (3) participants with non-painful DSPN; (4) participants with painful DSPN. For each variable, the largest, but still significant, *p* value is provided

NRDI, noradrenaline (norepinephrine)–dopamine reuptake inhibitors; NSAIDs, nonsteroidal anti-inflammatory drugs; SNRI, serotonin–noradrenaline (norepinephrine) reuptake inhibitors; SSRI, selective serotonin reuptake inhibitors

### Corneal confocal microscopy parameters

The values for all parameters for each group and the results of the statistical comparisons are summarised in Table 3. The most relevant findings are discussed below.

**Table 3:** Corneal confocal microscopy parameters for central cornea and inferior whorl, and axonal swelling and CSEMNs’ attributes

	No diabetes (*n*=27)	Diabetes but no DSPN (*n*=33)	Non-painful DSPN (*n*=25)	Painful DSPN (*n*=18)	*p* value (ANCOVA)	Adjusted* p* value	Pairwise comparisons
Central cornea							
CNFL (mm/mm^2^)	16.1±2.1	13.9±3.8	11.4±3.2	11.0±3.8	<0.001	≤0.024	1−2, 1−3, 1−4, 2−3, 2−4
CNBD (no./mm^2^)	38.0±14.1	33.5±16.0	22.5±12.3	27.0±22.8	0.0045	≤0.021	1−3, 2−3
CNFD (no./mm^2^)	27.5±6.2	23.2±7.3	17.9±5.9	16.7±7.2	<0.001	≤0.031	1−2, 1−3, 1−4, 2−3, 2−4
CTBD (no./mm^2^)	56.7±22.0	50.3±22.5	38.0±19.7	36.7±20.8	0.003	≤0.009	1−3, 1−4
CNFractalDimension	1.5±0.02	1.5±0.04	1.5±0.05	1.4±0.06	<0.001	≤0.032	1−3, 1−4, 2−3, 2−4
CNFW (mm/mm^2^)	0.0±0.0015	0.0±0.0012	0.0±0.0018	0.0±0.0022	0.42		
CNFA (mm/mm^2^)	0.0±0.0020	0.0±0.0018	0.0±0.0022	0.0±0.0014	0.085		
Inferior whorl							
IWL (mm/mm^2^)	14.7±4.1	14.4±4.0	9.4±4.4	10.7±5.5	<0.001	≤0.036	1−3, 1−4, 2−3
Combination of corneal metrics							
TNFL (mm/mm^2^)	30.9±5.3	28.3±7.2	20.7±6.3	21.7±8.9	<0.001	≤0.011	1−3, 1−4, 2−3, 2−4
ANFL (mm/mm^2^)	15.4±2.6	14.1±3.6	10.4±3.1	10.8±4.4	<0.001	≤0.012	1−3, 1−4, 2−3, 2−4
Ratio of CNFL to CNFractalDimension	10.7±1.3	9.4±2.3	7.8±1.9	7.5±2.4	<0.001	≤0.028	1−2, 1−3, 1−4, 2−3, 2−4
Presence of attributes							
Axonal swelling	0 (0)	3 (9.1)	7 (28.0)	13 (72.2)	<0.001	≤0.018	1−3, 1−4, 2−4, 3−4
Axonal distension	0 (0)	2 (6.1)	8 (32.0)	8 (44.4)	<0.001	≤0.042	1−3, 1−4, 2−3, 2−4
Enlarged bulges	1 (3.7)	2 (6.1)	1 (4.0)	5 (27.8)	0.038		
Hyper-reflective diffuse pattern	5 (18.5)	6 (18.2)	10 (40.0)	10 (55.6)	0.016		
Combination of all attributes	5 (18.5)	10 (30.3)	17 (68.0)	18 (100)	<0.001	≤0.026	1−3, 1−4, 2−3, 2−4, 3−4
Number of attributes							
Axonal swelling							
0	27 (100 )	30 (90.9)	18 (72.0)	5 (27.8)	<0.001	≤0.044	1−3, 1−4, 2−3, 2−4, 3−4
1	0 (0)	3 (9.1)	2 (8.0)	7 (38.9)			
2	0 (0)	0 (0)	3 (12.0)	6 (33.3)			
3	0 (0)	0 (0)	2 (8.0)	0 (0)			
4	0 (0)	0 (0)	2 (8.0)	0 (0)			
Axonal distension							
0	27 (100 )	31 (93.9)	17 (68.0)	11 (61.1)	<0.001	≤0.015	1−4, 2−4, 3−4
1	0 (0)	2 (6.1)	5 (20.0)	1 (5.6)			
2	0 (0)	0 (0)	2 (8.0)	3 (16.7)			
3	0 (0)	0 (0)	1 (4.0)	1 (5.6)			
4	0 (0)	0 (0)	0 (0)	2 (11.1)			
Enlarged bulges							
0	26 (96.3)	31 (93.9)	24 (96.0)	13 (72.2)	0.042		
1	1 (3.7)	1 (3.0)	0 (0)	3 (16.7)			
2	0 (0)	1 (3.0)	1 (4.0)	2 (11.1)			
3	0 (0)	0 (0)	0 (0)	0 (0)			
4	0 (0)	0 (0)	0 (0)	0 (0)			
Hyper-reflective diffuse pattern							
0	22 (81.5)	27 (81.8)	15 (60.0)	8 (44.4)	0.003		
1	5 (18.5)	5 (15.2)	5 (20.0)	7 (38.9)			
2	0 (0)	1 (3.00)	5 (20.0)	2 (11.1)			
3	0 (0)	0 (0)	0 (0)	1 (5.6)			
4	0 (0)	0 (0)	0 (0)	0 (0)			

Continuous data are means ± SD and were analysed by one-way ANCOVA with post hoc Bonferroni–Holm tests. Categorical data are *n* (%) and were analysed using Fisher’s exact test with pairwise tests of independence using the Holm method to adjust for multiple comparisons.

The column ‘*p* value ANCOVA’ shows the *p* value for the global comparison across all four groups for each variable. The column ‘Adjusted *p* value’ shows the *p* value for the pairwise comparisons between group pairs as indicated in the final column: (1) participants without diabetes; (2) participants with diabetes but without DSPN; (3) participants with non-painful DSPN; (4) participants with painful DSPN. For each variable, the largest, but still significant, *p* value is provided

#### Nerve morphological metrics

**Fig. 4:**
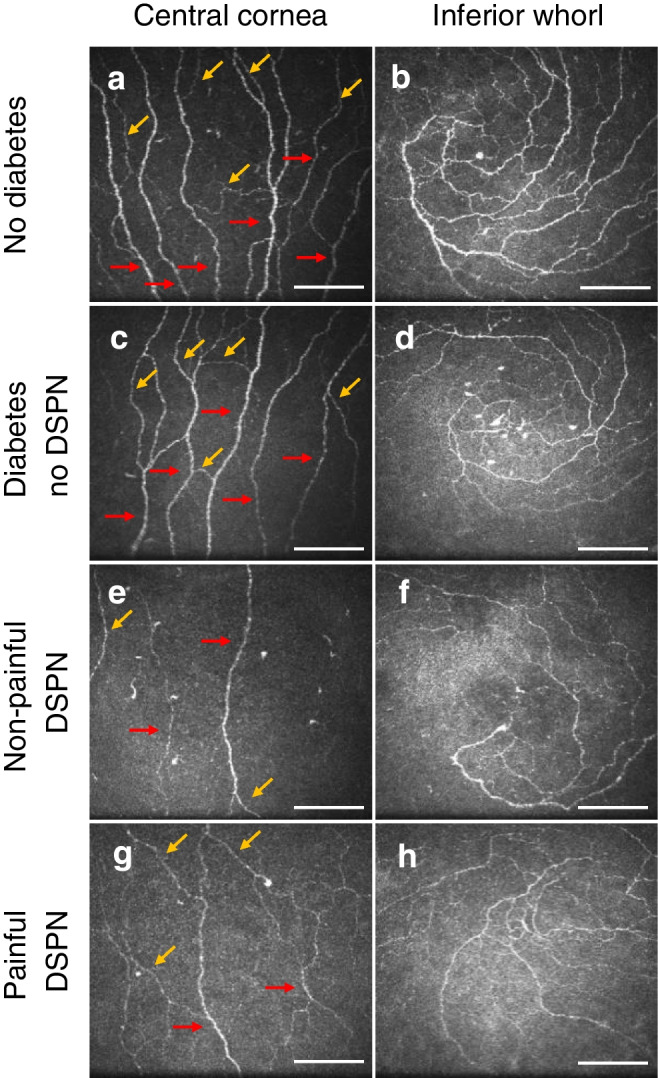
Representative IVCM images from the central cornea (**a**, **c**, **e**, **g**) and the inferior whorl (**b**, **d**, **f**, **h**). Images are representative for participants without diabetes (**a**, **b**), participants with diabetes but without DSPN (**c**, **d**), participants with non-painful DSPN (**e**, **f**) and participants with painful DSPN (**g**, **h**). The red arrows in (**a**, **c**, **e** and **g**) indicate main nerve fibres (to calculate CNFD), and the yellow arrows indicate branch fibres (to calculate CNBD). In the central cornea and the inferior whorl, successive loss of nerve fibre density may be observed from the participant without diabetes to the participant with painful DSPN. Scale bar, 100 µm

**Fig. 5:**
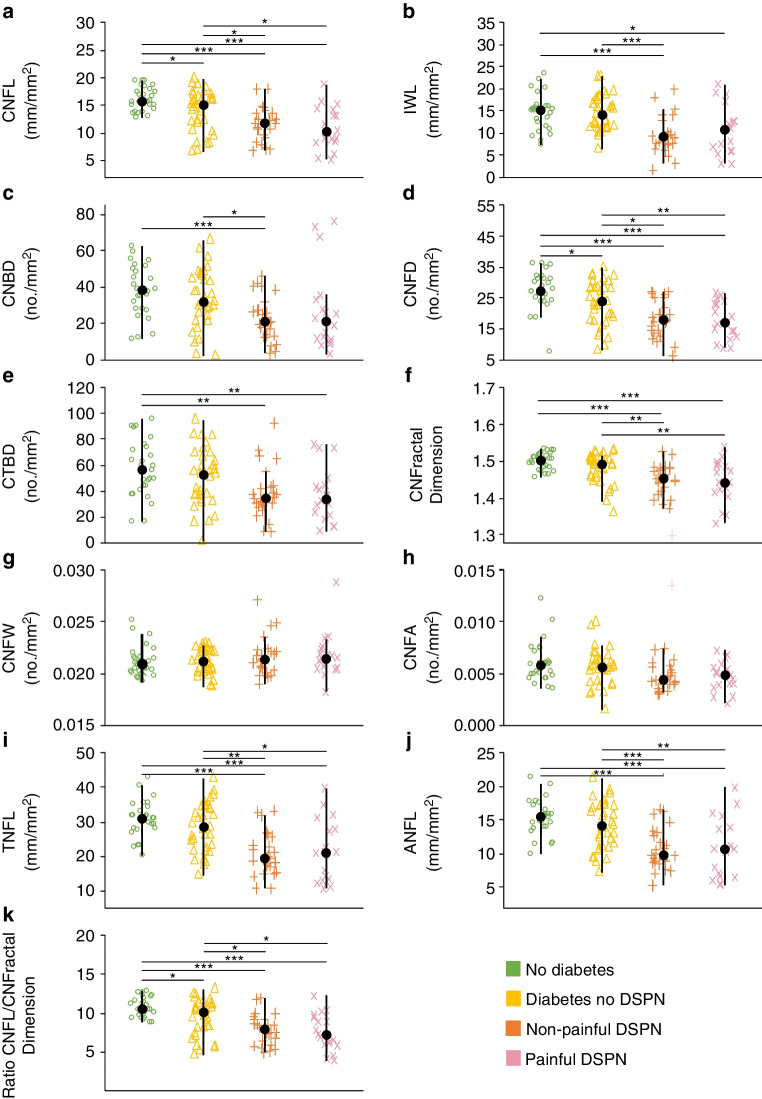
Boxplots for the corneal confocal microscopy parameters from the central cornea (**a**, **c**–**h**) and the inferior whorl (**b**), and for the combined metrics (**i**–**k**). Significant differences determined using post hoc tests with Bonferroni correction are indicated (*p<0.05; **p<0.01; ***p<0.001)

Representative images of the nerve morphology for each group are illustrated in Fig. 4. The findings and statistical comparisons are summarised in Fig. 5 and Table 3. There were no differences in any parameter in the central cornea, the inferior whorl or the combined metrics that indicated a significant difference between participants with painful DSPN and those with non-painful DSPN (*p*≥0.58, Fig. 5a–k). At the central cornea, CNFW and CNFA showed no significant differences (all *p*≥0.085; Fig. 5g,h). All other IVCM parameters were reduced in participants with painful DSPN compared to participants without diabetes (*p*≤0.009) with the exception of CNBD (*p*=0.053; Fig. 5a,d–f). Additionally, IVCM parameters were reduced in participants with painful DSPN compared to participants with diabetes but without DSPN (*p*≤0.032), with the exception of CNBD (*p*=0.25) and CTBD (*p*=0.083; Fig. 5a,d–f). In participants with non-painful DSPN, there was a reduction in IVCM parameters compared to participants with diabetes but without DSPN (*p*≤0.032; except for CTBD for which *p*=0.083) and compared to participants without diabetes (*p*≤0.009; Fig. 5a,c–f). CNFL (*p*=0.025) and CNFD (*p*=0.031) were reduced in participants with diabetes but without DSPN compared to participants without diabetes (Fig. 5a,d).

At the inferior whorl, IWL was reduced in participants with painful DSPN compared to participants without diabetes (*p*=0.036), and between participants with non-painful DSPN and participants with diabetes but without DSPN (*p*=0.005) and between participants with non-painful DSPN and participants without diabetes (*p*=0.002) (Fig. 5b).

For the combined metrics, no differences were observed in the ratio CNFL/CNFractalDimension, ANFL and TNFL between participants with painful DSPN and participants with non-painful DSPN (*p*≥0.60; Fig. 5i–k). The ratio CNFL/CNFractalDimension (*p*=0.028) but not ANFL (*p*=0.28) or TNFL (*p*=0.28) was reduced in participants with diabetes but without DSPN compared to participants without diabetes (Fig. 5i–k). All other group comparisons showed a decrease of ANFL (*p*≤0.012), TNFL (*p*≤0.011) and CNFL/CNFractalDimension (*p*≤0.028) across groups (Fig. 5i–k).

In the sensitivity analysis, parameters at the central cornea were not different between participants with painful DSPN and those with pain-free DSPN (*p*=1). No differences were found between participants with pain-free DSPN and participants with diabetes but without DSPN for any IVCM parameters at the central cornea (*p*≥0.077). Compared to participants without diabetes, CNFD, CNBD, CNFL, CTBD and CNFractalDimension were significantly reduced in the pain-free DSPN group (*p*≤0.012). At the inferior whorl, the pain-free DSPN group showed a larger reduction in IWL compared to participants without diabetes (*p*=0.010) and participants with diabetes but without DSPN (*p*=0.021). For the combined metrics, the pain-free group showed a reduction in ANFL and TNFL compared to participants without diabetes (*p*≤0.0065) and participants with diabetes but without DSPN (*p*≤0.035). Additionally, the ratio CNFL/CNFractalDimension was reduced in the pain-free group compared to participants without diabetes (*p*<0.001). The values for all parameters and groups in the sensitivity analysis are summarised in ESM Table 1.

#### Axonal swelling

**Fig. 6:**
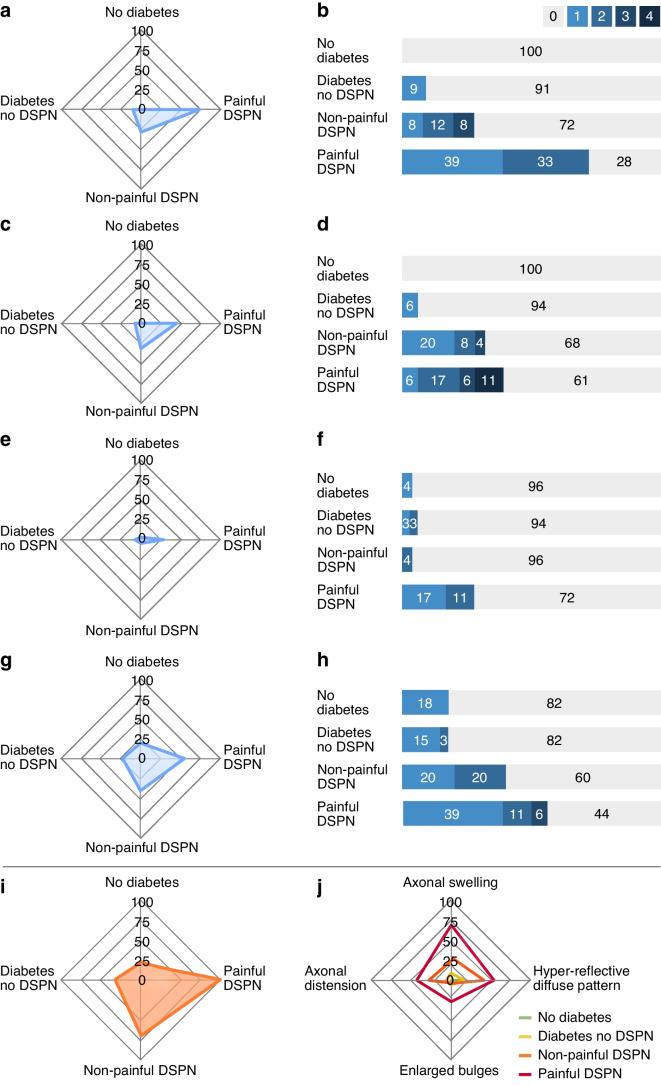
Axonal swelling and CSEMNs for the various groups. Radar plots illustrate the proportions of participants with axonal swelling (**a**) and CSEMNs (**c**, **e**, **g**), presence of all CSEMNs and axonal swelling combined per group (**i**) and presence of each CSEMNs and axonal swelling per group (**j**). Numbers 0–100 refer to per cent (%). Bar charts show the frequency of axonal swelling (**b**) and CSEMNs (**d**, **f**, **h**). Axonal swelling, axonal distention and enlarged bulges are considered pathological, whereas hyper-reflective diffuse patterns may be considered normal but are more frequent in participants with DSPN

Figure 6a shows the presence of axonal swellings for each group. Axonal swelling was more prevalent in participants with painful DSPN compared to participants with non-painful DSPN (*p*=0.018), participants with diabetes but without DSPN (*p*<0.001) and participants without diabetes (*p*<0.001). Additionally, axonal swelling was more frequently observed in participants with non-painful DSPN compared to participants without diabetes (*p*=0.014).

Figure 6b shows the frequency of axonal swelling for each group. A higher number of axonal swellings was observed in participants with painful DSPN compared with non-painful DSPN (*p*=0.03), participants with diabetes but without DSPN (*p*<0.001) and participants without diabetes (*p*<0.001). Additionally, the number of axonal swellings was higher in participants with non-painful DSPN compared to those with diabetes but without DSPN (*p*=0.044) and participants without diabetes (*p*=0.023).

#### Corneal sub-epithelial microneuromas

The presence of CSEMNs is presented for each group in Fig. 6c,e,g. Axonal distension (Fig. 6c) was more prevalent in participants with painful DSPN compared to participants with diabetes but without DSPN (*p*=0.008) and participants without diabetes (*p*=0.001), and in participants with non-painful DSPN compared to participants with diabetes but without DSPN (*p*=0.042) and participants without diabetes (*p*=0.007). There was an overall difference between groups for the presence of enlarged bulges (Fig. 6e) and hyper-reflective diffuse patterns (Fig. 6g) (*p*≤0.038). Although the hyper-reflective diffusion pattern may be considered physiological [16], its prevalence differed between groups (*p*=0.016). Pairwise comparisons for the presence of enlarged bulges or a hyper-reflective diffuse pattern revealed no differences between groups.

For the frequency of CSEMNs (Fig. 6d,f,h), the number of axonal distensions was increased in participants with painful DSPN compared with all other groups (all *p*≤0.015). Although the ANCOVA analysis showed that the number of enlarged bulges (*p*=0.042) and hyper-reflective diffuse patterns (*p*=0.003) were significantly different, pairwise comparisons did not reveal any differences between groups.

The sensitivity analysis showed no differences between painful and pain-free DSPN participants for any of the CSEMN attributes (*p*≥0.080). Axonal distension was more common in participants in the pain-free DSPN group compared to participants without diabetes (*p*=0.018).

#### Axonal swelling and microneuromas combined

The presence of axonal swelling and all microneuromas combined was increased in participants with painful DSPN compared with all other groups (*p*≤0.026; Fig. 6i). There was a trend towards an increased presence of this combination of features in more severe groups (*p*≤0.022). No difference was detected between participants without diabetes and participants with diabetes but without DSPN (*p*=0.38).

## Discussion

The most important finding of this study arose from the comparison of axonal swelling and CSEMNs between the four groups. The presence of axonal swelling was increased in participants with painful DSPN compared with all other groups, and an increased frequency of axonal swelling and axonal distension was observed in participants with painful DSPN compared with all other groups. Although the number of hyper-reflective diffuse patterns and enlarged bulges increased across the groups, no differences were detected between participants with painful and non-painful DSPN.

Skin biopsies have suggested that axonal swellings are an early indicator of axon degeneration, a predictor of nerve fibre loss [36] and a possible pain generator [37] in painful neuropathies of the skin. Axonal swelling has been related to defective axonal transport, which commonly occurs in sensory neuropathy, and to the potential enhancement of mechanical and thermal sensitivity that leads to allodynia and hyperalgesia [32]. Our results revealed that axonal swelling was more frequent in participants with painful DSPN compared with all other groups, including non-painful DSPN. When exploring epidermal axonal swelling in participants with diabetes using skin biopsies [32, 37], higher epidermal axonal swelling ratios occurred more frequently in participants with diabetes compared with controls, and were either equally frequent [37] or more frequent [32] in participants with painful DSPN compared to those with non-painful DSPN [37]. An important difference with these skin biopsy results was their definition of axonal swelling (ratio of number of swellings to the number of fibres [36] vs frequency in our study). Due to the study design, we can only speculate that axonal swelling in the cornea may be related to the presence of pain in DSPN.

Interestingly, axonal swelling and axonal distension were only observed in participants with diabetes. The presence of axonal distension may reflect the effect of diabetes on neural tissues, and a higher number of distensions may be related to a more severe condition and even the presence of pain. Additionally, there was an increase in the number of axonal swellings and all CSEMNs across the groups, which may be related to the increasing severity of the clinical presentation. Hyper-reflective diffuse patterns were previously considered a normal, physiological finding as they reflect nerve penetration sites at the stromal–epithelial level and may appear dysmorphic when imaged using IVCM [16]. Hyper-reflective diffuse patterns were present in all groups; however, there was an overall increase in the number of hyper-reflective diffuse patterns across groups, from participants without diabetes to participants with painful DSPN.

Corneal nerve loss was more severe in participants with non-painful and painful DSPN compared to participants with diabetes but without DSPN or participants without diabetes. Nine of the 11 parameters assessed in the central cornea and inferior whorl showed a significant decline across groups, from participants without diabetes to participants with painful DSPN. These results are in line with previous studies that showed a deterioration in corneal nerve fibre length and density, and in the combined metrics, in the presence of DSPN compared with participants without DSPN or participants without diabetes [7, 30, 31, 38].

No differences were observed in any of the traditionally assessed corneal nerve morphological parameters between the painful and non-painful DSPN groups. Previously, a reduction in corneal nerve density and length, including in the whorl, was observed in participants with painful DSPN compared to those with non-painful DSPN [23, 39]. It is important to consider that participants in the non-painful DSPN group in our study may present with mild pain (i.e. a score on the NRS <4 does not necessarily indicate absence of pain) [22]. However, the cut-off that we used to differentiate painful from non-painful DSPN is common in corneal confocal studies [7, 22–24]. To address this limitation, we performed a sensitivity analysis including only pain-free participants (score on NRS = 0). Interestingly, the pain-free group did not differ from participants with diabetes but without DSPN at the central cornea, but IWL was reduced. This in line with a previous study in which IWL indicated an abnormality even in patients without DSPN [38].

An important limitation of this study is that the groups could not be balanced for relevant characteristics. Participants with diabetes but without DSPN were younger, had predominantly type 2 diabetes, had had diabetes for fewer years, and their HbA_1c_ levels were lower compared with the diabetes population reported in other studies [7, 38, 40]. These differences were due to difficulties in recruitment during the COVID-19 pandemic. A longer duration of diabetes, higher HbA_1c_ levels and age have been associated with reductions in IVCM parameters (e.g. CNFL) in type 2 diabetes, whereas only the duration of diabetes appears to influence CNFL in type 1 diabetes [41]. Future studies should attempt to balance the distribution of type 1 and type 2 diabetes across groups. The groups of patients with non-painful and painful DSPN were no different in terms of HbA_1c_ levels and in years lived with DSPN. However, HbA_1c_ levels were higher in participants with painful and non-painful DSPN compared to participants with diabetes but without DSPN. Controlling for HbA_1c_ levels across groups is difficult, as higher HbA_1c_ levels are associated with increased risk of DSPN [42]. Finally, sample size was calculated based on previous results for corneal nerve fibre parameters [34] due to the exploratory nature of this study. Future studies are encouraged to calculate adequate sample sizes for studies of CSEMN presence and frequency.

Although the presence and frequency of axonal swelling and CSEMNs were identified as described in previous publications [11, 14], these measures are novel. Due to the nature of the confocal microscopy acquisition, it cannot be ruled out, but is unlikely, that immature dendritic cells (e.g. globular-shaped dendritic cells without dendritic processes) [43] have been mistaken for CSEMNs, and, more specifically, axonal distension. However, as the investigator assessing axonal swelling and CSEMNs was blinded to the participant’s group allocation, we have no reason to assume that systematic errors were made between groups.

In conclusion, IVCM offers non-invasive and rapid evaluation of the pathophysiology behind DSPN. The degenerative processes involved were reflected in the detection of fibre loss that occurs in DSPN. Interestingly, use of microscopy to detect the presence and frequency of axonal swelling and axonal distension may reveal individual pathomechanisms in people with (painful) DSPN. Specifically, the presence of axonal swelling may be indicative of the regeneration processes in DSPN that have been linked to the presence of pain.

### Supplementary Information

Below is the link to the electronic supplementary material.

Supplementary file1 (PDF 187 KB)

## Data Availability

The datasets generated and/or analysed during the current study are available from the corresponding author on reasonable request.

## Abbreviations

ANFL: Average nerve fibre length
CNBD: Corneal nerve branch density
CNFA: Corneal nerve fibre area
CNFD: Corneal nerve fibre density
CNFL: Corneal nerve fibre length
CNFractalDimension: Corneal nerve fractal dimension
CNFW: Corneal nerve fibre width
CSEMN: Corneal sub-epithelial microneuroma
CTBD: Corneal fibre total branch density
DIANE: DIAbetic NEuropathy Project
DSPN: Distal symmetrical polyneuropathy
IVCM: In vivo corneal confocal microscopy
IWL: Inferior whorl length
NRS: Numerical rating scale
TNFL: Total nerve fibre length
