# Correction to: Occurrence of corneal sub-epithelial microneuromas and axonal swelling in people with diabetes with and without (painful) diabetic neuropathy

**DOI:** 10.1007/s00125-024-06138-z

**Published:** 2024-04-05

**Authors:** Eva Sierra‑Silvestre, Ricardo J. Andrade, Luisa H. Colorado, Katie Edwards, Michel W. Coppieters

**Affiliations:** 1 Menzies Health Institute Queensland, Griffith University, Brisbane, QLD Australia; 2 School of Health Sciences and Social Work, Griffith University, Brisbane, QLD Australia; 3 Amsterdam Movement Sciences – Musculoskeletal Health Program, Faculty of Behavioural and Movement Sciences, Vrije Universiteit Amsterdam, Amsterdam, the Netherlands; 4 Movement – Interactions – Performance (MIP), Nantes University, Nantes, France; 5 Centre for Vision and Eye Research, School of Optometry and Vision Science, Queensland University of Technology, Brisbane, Australia

**Correction to: Diabetologia**

10.1007/s00125-023-05945-0

Unfortunately, Luisa H. Colorado’s name appeared incorrectly. The original article has been corrected.

