# Supplementary material for: Occurrence of corneal sub-epithelial microneuromas and axonal swelling in people with diabetes with and without (painful) diabetic neuropathy
Source: Diabetologia. 2023 Jun 10;66(9):1719–34. doi: 10.1007/s00125-023-05945-0 (PMC10257488; doi:10.1007/s00125-023-05945-0)
Supplement: Supplementary file 1 — Supplementary file1 (PDF 187 KB) [file 125_2023_5945_MOESM1_ESM.pdf]

ELECTRONIC SUPPLEMENTARY MATERIAL

Methods

Statistical analysis

Several covariates were explored in our analysis, which included HbA_{1c} levels, duration of diabetes, and duration of DSPN into our ANCOVA analysis. Independence of the covariate from the independent variable (groups) is an important assumption for ANCOVA [1]. In this study, we could not add these variables as covariates as they were not independent from the groups. When the groups in the independent variable differ on the covariate (e.g., people with diabetes with DSPN will have higher HbA_{1c} levels than people without diabetes), putting the covariate into the analysis will not 'control for' or 'balance out' those differences. We could not randomise participants into experimental groups as group allocation was based on the diagnosis of diabetes, which relied on the HbA_{1c} level, and the presence of painful or non-painful DSPN.

Corneal confocal microscopy parameters

All participants underwent an examination of the sub-basal plexus using a Heidelberg Tomograph Rostock Cornea Module III (Heidelberg Engineering GmbH, Heidelberg, Germany), according to published methods [2]. The measures collected included:

- Central cornea
    - Corneal nerve fibre density (CNFD, total number of main nerves per square millimetre; no./mm^2)
    - Corneal nerve branch density (CNBD, total number of branches per square millimetre; no./mm^2)
    - Corneal nerve fibre length (CNFL, total length of main nerves and nerve branches per square millimetre; mm/mm^2)
    - Corneal nerve fibre area (CNFA, total nerve fibre area per square millimetre; mm/mm^2)
    - Corneal fibre total branch density (CTBD, total number of branch points per square millimetre; no./mm^2)
    - Corneal nerve fibre width (CNFW, the average nerve fibre width per square millimetre; mm/mm^2)
    - Corneal nerve fractal dimension (CNFractalDimension, a measure of nerve complexity obtained by the ratio of the change in detail to the change in scale in a IVCM image)
- Inferior whorl
    - Inferior whorl length (IWL, total length of nerves per square millimetre; mm/mm^2)
- Combination of metrics
    - Ratio CNFL/CNFractalDimension
    - Average nerve fibre length (ANFL, CNFL + IWL/2) (mm/mm^2)
    - Total nerve fibre length (TNFL, CNFL + IWL) (mm/mm^2) [3].

ESM TABLE 1

ESM Table 1. Sensitivity analysis restricted to pain-free DSPN participants showing corneal confocal microscopy parameters for the central cornea and inferior whorl, and attributes of the corneal epithelial microneuromas (CEMNs).

	Healthy participants (n=27)	Diabetes no DSPN (n=33)	Pain-free DSPN (n=11)	Painful DSPN (n=18)	p-value
Nerve morphology					
Central cornea					
CNFD (no./mm^2)	27.5 ±6.2	23.2 ±7.3	18.3 ±6.0	16.7 ±7.2	<0.001[1-3,1-4,2-4]
CNBD (no./mm^2)	38.0 ±14.1	33.5 ±16.0	20.6 ±9.8	27.0 ±22.8	0.018[1-3]
CNFL (mm/mm^2)	16.1 ±2.1	13.9 ±3.8	11.1 ±3.1	11.0 ±3.8	<0.001[1-3,,1-4,2-4]
CTBD (no./mm^2)	56.7 ±22.0	50.3 ±22.5	34.2 ±13.0	36.7±20.8	0.003[1-3,1-4]
CNFA (mm/mm^2)	0.0 ±0.002	0.0 ±0.002	0.005 ±0.001	0.005 ±0.001	0.052
CNFW (mm/mm^2)	0.0 ±0.002	0.0 ±0.001	0.02 ±0.001	0.02 ±0.002	0.55
CNFractalDimension	1.5 ±0.02	1.5 ±0.05	1.4 ±0.04	1.4 ±0.06	<0.001[1-3,1-4,2-4]
Inferior whorl					
IWL (mm/mm^2)	14.7 ±4.1	14.4 ±4.0	9.1 ±3.3	10.7 ±5.5	0.002 [1-3,2-3]
Combination of corneal metrics					
Ratio CNFL/CNFractalDimension	10.7 ±1.3	9.4 ±2.3	7.6 ±1.9	7.5 ±2.4	<0.001 [1-3,1-4,2-4]
ANFL (mm/mm^2)	15.4 ±2.6	14.1 ±3.6	10.4 ±3.3	10.8± 4.4	<0.001 [1-3,1-4,2-3,2-4]
TNFL (mm/mm^2)	30.9 ±5.3	28.3 ±7.2	19.4 ±6.0	21.7 ±8.9	<0.001 [1-3,1-4,2-3,2-4]
Microneuroma attributes					
Axonal swelling	0 (0%)	3 (11.1%)	3 (27 %)	13 (72 %)	<0.001 [1-4,2-4]
Axonal distension	0 (0%)	2 (7.4%)	4 (36 %)	8 (44 %)	<0.001 [1-3,1-4,2-4]
Enlarged bulges	1 (3.7%)	2 (7.4%)	0 (0 %)	5 (28 %)	0.039
Hyperreflective diffuse pattern	5 (18.5%)	6 (22.2%)	5 (45 %)	10 (56 %)	0.014

Data shown as mean (SD) and analysed by one-way ANCOVAs, with post hoc tests using Bonferroni-Holm correction (pairwise comparison). Categorical data are reported as frequencies (percentages) with Fisher's exact test with pairwise tests of independence. Pairwise differences indicate significant *p* values between group pairs: 1: Healthy participants; 2: diabetes no DSPN; 3: Non-painful DSPN; 4: Painful DSPN. DSPN: distal symmetrical polyneuropathy; CNFD: corneal nerve fibre density; CNBD, corneal nerve branch density; CNFL, corneal nerve fibre length; CNFA, corneal nerve fibre area; CTBD, corneal fibre total branch density; CNFW, corneal nerve fibre width; CNFractalDimension, corneal nerve fractal dimension; IWL, inferior whorl length; ANFL, average nerve fibre length of CNFL and IWL; TNFL, total nerve fibre length, sum of CNFL and IWL

References

1. Field, A., Miles, J., & Field, Z. (2012). Discovering statistics using R. SAGE Publications.
2. Vagenas D, Pritchard N, Edwards K, et al (2012) Optimal image sample size for corneal nerve morphometry. Optom Vis Sci 89(5):812–817. https://doi.org/10.1097/OPX.0b013e31824ee8c9
3. Kalteniece A, Ferdousi M, Petropoulos I, et al (2018) Greater corneal nerve loss at the inferior whorl is related to the presence of diabetic neuropathy and painful diabetic neuropathy. Sci Rep 8(1):3283. https://doi.org/10.1038/s41598-018-21643-z
